# Borderline Personality Disorder: A Comprehensive Review of Current Diagnostic Practices, Treatment Modalities, and Key Controversies

**DOI:** 10.7759/cureus.75893

**Published:** 2024-12-17

**Authors:** Saif Azzam, Rahma Almari, Karees Khattab, Ammar Badr, Arwa R Balawi, Rana Haddad, Rawan Almasri, Giustino Varrassi

**Affiliations:** 1 Clinical Sciences, Yarmouk University, Irbid, JOR; 2 Medicine, Jordan University of Science and Technology, Irbid, JOR; 3 Medicine, University of Jordan, Irbid, JOR; 4 Psychiatry, International Medical Corps, Amman, JOR; 5 Pain Medicine, Fondazione Paolo Procacci, Rome, ITA

**Keywords:** borderline personality disorder, dialectical behavior therapy, mentalization-based treatment, schema-focused psychotherapy, transference-focused psychotherapy

## Abstract

Borderline personality disorder (BPD) is a complex psychiatric condition characterized by pervasive patterns of instability in emotions, interpersonal relationships, and self-image. This comprehensive review explores the current diagnostic practices, treatment modalities, and ongoing controversies surrounding BPD. We discuss established and proposed diagnostic criteria, highlight the limitations of current assessment tools, and examine the epidemiology of the disorder, including its prevalence and comorbidities. The effectiveness of psychotherapeutic approaches such as dialectical behavior therapy, mentalization-based treatment, transference-focused psychotherapy, and schema-focused psychotherapy is evaluated alongside the role of pharmacological interventions. Furthermore, we address critical controversies, including misdiagnosis, the impact of trauma, stigma, and the ongoing debate regarding the treatability and recovery potential for individuals with BPD. By synthesizing these facets, we aim to provide a nuanced understanding of BPD and inform future research and clinical practice.

## Introduction and background

Borderline personality disorder (BPD) is a complex and often misunderstood mental health condition characterized by pervasive patterns of instability in emotional regulation, interpersonal relationships, self-image, and behavior [1]. Recognized for its significant impact on individuals and their loved ones, BPD affects approximately 1-2% of the general population and is prevalent in clinical settings, particularly in psychiatric and therapeutic environments. The disorder manifests through a range of symptoms, including intense emotional responses, impulsive behavior, and chronic feelings of emptiness or abandonment [2].

The origins of BPD are multifaceted, shaped by an interplay of genetic predispositions, biological vulnerabilities, and environmental influences [2]. Childhood trauma, particularly adverse experiences such as physical, emotional, or sexual abuse, neglect, and inconsistent caregiving, has been extensively linked to the development of BPD. These early disruptions impair attachment processes and emotional regulation, contributing to the interpersonal and affective instability characteristic of the disorder. This dynamic interplay between inherited and environmental factors highlights the complexity of BPD, emphasizing that its onset and progression result from a convergence of diverse influences rather than any single causative element [2].

Historically, BPD was viewed as a borderline state between neurosis and psychosis, first identified in 1938 and often labeled as an untreatable condition [3,4]. However, recent advancements in therapeutic approaches, particularly dialectical behavior therapy (DBT), have transformed this narrative [3,4]. DBT, developed specifically for individuals with BPD, emphasizes skills such as emotional regulation, distress tolerance, interpersonal effectiveness, and mindfulness [3,4]. Empirical evidence supports the efficacy of DBT, demonstrating significant reductions in self-harming behaviors and improvements in emotional regulation. This shift in understanding underscores the potential for recovery and the importance of a compassionate, evidence-based approach to treatment [3,4].

In addition to psychotherapeutic interventions, understanding the diagnostic challenges associated with BPD is crucial. Misdiagnosis often occurs due to overlapping symptoms with other mental health conditions, such as bipolar disorder and anxiety disorders. Such challenges can lead to ineffective treatment plans and exacerbate the individual’s struggles. Thus, clinicians must adopt a culturally competent perspective that acknowledges the diversity of symptom expression and the unique experiences of individuals with BPD [5].

Overall, a nuanced understanding of BPD is essential for effective diagnosis and treatment. Acknowledging the complexities of its etiology, symptomatology, and therapeutic approaches can foster better outcomes for those navigating the challenges of this disorder, ultimately paving the way for enhanced recovery and improved quality of life [6-8].

The purpose of this review is to provide a comprehensive examination of current diagnostic practices, treatment modalities, and key controversies surrounding BPD. By synthesizing the latest research and clinical insights, this review aims to enhance understanding of the disorder, address common misconceptions, and inform best practices in the diagnosis and treatment of BPD. Through a thorough exploration of various therapeutic approaches and ongoing debates within the field, this review aspires to contribute to improved outcomes for individuals living with BPD.

## Review

Clinical presentation of BPD

BPD is characterized by pervasive patterns of instability in emotional regulation, self-image, interpersonal relationships, and behavior. Core features include frantic efforts to avoid abandonment, intense and unstable relationships fluctuating between idealization and devaluation, and a distorted sense of self. Individuals often display impulsive behaviors in potentially harmful areas, recurrent suicidal or self-harming tendencies, and mood reactivity with episodic dysphoria, irritability, or anxiety. Chronic feelings of emptiness, difficulty managing intense anger, and transient stress-related paranoia or dissociation further complicate the presentation [6,9].

Epidemiology

BPD is a significant mental health condition affecting about 1% to 2% of the general population, with much higher rates of 15% to 20% in psychiatric settings [10]. Women are disproportionately affected, with an approximate female-to-male ratio of 3:1, although this ratio is influenced by the higher prevalence of women in outpatient settings, which may not fully represent the general population [11,12]. BPD frequently co-occurs with other psychiatric disorders such as mood, anxiety, and substance use disorders, which complicates diagnosis and treatment [13]. Comorbidities often increase symptom severity and raise suicide risk; around 10% of those with BPD may die by suicide [2,14]. Symptoms typically begin in late adolescence or early adulthood, although diagnosis is often delayed due to stigma and symptom overlap with other conditions [15]. Epidemiological insights into BPD are essential for improving prevention, intervention, and reducing societal stigma [16].

Pharmacology

Pharmacological treatments in BPD primarily target specific symptoms rather than the core disorder itself. Due to the difficulty in addressing BPD's central characteristics, such as emotional dysregulation, impulsivity, and unstable interpersonal relationships, pharmacotherapy is typically used as an adjunct to psychotherapy, particularly DBT [17]. Guidelines from the National Institute for Health and Care Excellence and the American Psychiatric Association emphasize that medications, such as antidepressants, mood stabilizers, and antipsychotics, are prescribed to manage symptoms such as depression, impulsivity, and mood instability, rather than to treat the disorder directly [18,19].

Antidepressants

Antidepressants and mood stabilizers are common adjuncts in treating BPD, primarily to manage co-occurring symptoms, such as mood instability, impulsivity, and aggression, or to treat coexisting Axis I disorders, such as depression and anxiety. Selective serotonin reuptake inhibitors (SSRIs), such as fluoxetine, are often prescribed to address depressive symptoms in BPD. While SSRIs are effective for mood disorders, they show limited benefits for core BPD symptoms. For example, a study by Simpson et al. [20] on BPD patients receiving DBT found no significant improvement when fluoxetine was added [20,21]. Another study by Coccaro et al. [22] demonstrated that fluoxetine reduced impulsive aggression in personality disorders, though its impact on fundamental personality traits was less evident. Antidepressants mainly target comorbid mood or anxiety disorders rather than core BPD symptoms such as emptiness [23].

Mood Stabilizers

Mood stabilizers, including lithium, lamotrigine, and carbamazepine, address impulsivity, anger, and emotional dysregulation in BPD. Lithium, commonly used for bipolar disorder, may reduce impulsivity but carries risks such as thyroid dysfunction [24,25]. Lamotrigine is shown to improve emotional regulation with fewer side effects, while carbamazepine is considered particularly effective for managing affective instability and impulsive aggression [26-29]. Mood stabilizers are generally more beneficial when combined with psychotherapy, providing a foundation for emotional control that therapeutic techniques can further enhance [30].

Antipsychotics

Atypical antipsychotics, including aripiprazole, quetiapine, and olanzapine, are frequently used in treating BPD to help manage symptoms such as impulsivity, transient psychosis, and mood dysregulation. Aripiprazole has shown benefits in improving impulse control and reducing anger, while olanzapine is useful for severe mood instability, though it can lead to weight gain and metabolic issues [23,24]. Quetiapine, particularly in low doses, is effective for easing anxiety and dysphoria, making it beneficial for BPD patients with anxiety-related symptoms [31]. While antipsychotics can relieve symptoms, they are prescribed with caution due to potential side effects and are primarily used for short-term symptom management rather than addressing core BPD traits directly [32,33].

Psychotherapy

Psychotherapy is essential in treating BPD, tackling the complex emotional and relational challenges of the disorder. Therapeutic approaches such as DBT, mentalization-based treatment (MBT), and transference-focused psychotherapy (TFP) have proven effective in fostering emotional regulation, improving relationships, and building a stable sense of self. Each modality provides unique, targeted strategies suited to BPD's intricacies, highlighting the need for personalized treatment. In the following sections, we will examine these therapies, assessing their effectiveness and limitations in managing BPD symptoms [34,35].

DBT

What is DBT?

Developed by Marsha Linehan, DBT combines cognitive-behavioral techniques with strategies for acceptance, aiming to balance change with validation for patients, especially those with high-risk behaviors and BPD [19,36]. Recognized by major health guidelines, DBT includes individual therapy, group skills training, and therapist consultations [19,36].

Efficacy of DBT

Studies reveal DBT’s impact on reducing suicidality, self-harm, and depressive symptoms, with effects lasting up to two years after treatment. The Berlin borderline study also found that 77% of participants no longer met BPD diagnostic criteria after one year of DBT, showcasing significant improvements in BPD symptoms [37,38].

Limitations of DBT

DBT’s limitations include reduced effectiveness for severe cases, challenges in long-term efficacy, and comparable outcomes with other therapies, such as general psychiatric management. Some patients find DBT confrontational due to the emphasis on interpreting negative emotions toward the therapist, leading to higher dropout rates. Additionally, methodological concerns such as small sample sizes limit the generalizability of findings, suggesting DBT’s benefits may not apply universally across diverse populations [39-43].

MBT

What is MBT?

Developed by Peter Fonagy and Anthony Bateman, MBT is a psychodynamic therapy for BPD, rooted in attachment and cognitive theory. It helps patients enhance mentalization-the ability to understand their own and others' mental states-aiming to improve emotion regulation, impulse control, and relationships. Cost effective when delivered by general mental health professionals, MBT has shown positive, lasting effects for BPD patients in both partial hospitalization and outpatient settings [4,44-46].

Efficacy of MBT

An eight-year follow-up study found MBT significantly reduced BPD symptoms, self-harm, and service utilization compared to treatment as usual, underscoring its efficacy as a long-term therapy [47].

Limitations of MBT 

MBT may be less effective for patients with severe trauma or comorbid conditions, such as substance use disorders, which complicate the mentalization process. While MBT aids mentalizing abilities, it may not fully address emotional dysregulation and impulsivity, suggesting a need for integration with other therapies for comprehensive treatment [47-49].

Schema therapy (ST)

*What is ST​*​​​​​​*?*

**Figure 1:**
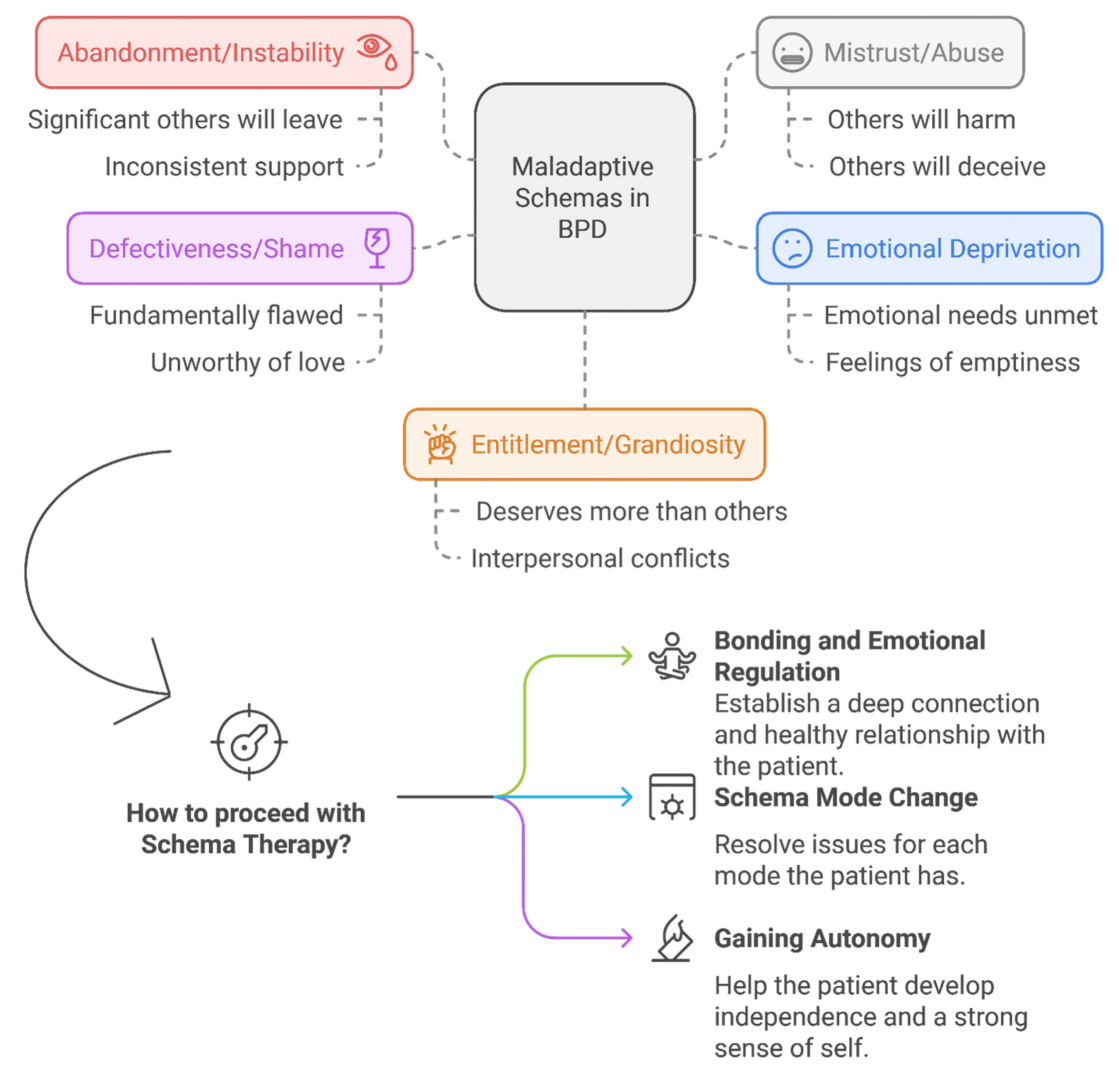
Overview of maladaptive schemas in BPD and the approach to schema therapy. The diagram presents core maladaptive schemas frequently seen in BPD, such as abandonment/instability, defectiveness/shame, entitlement/grandiosity, emotional deprivation, and mistrust/abuse. Each schema encompasses underlying beliefs, such as fear of abandonment or feelings of unworthiness. Schema therapy involves three main therapeutic strategies: bonding and emotional regulation, focusing on building a secure therapeutic relationship and helping regulate emotions; schema mode change, which targets maladaptive schema modes to resolve specific issues; and gaining autonomy, which fosters independence and self-identity. Image credits: Saif Azzam and Rahma Almari BPD, Borderline personality disorder

Developed by Jeff Young in 1990, ST was designed to treat patients with rigid cognitive patterns who did not respond well to cognitive-behavioral therapy. ST is particularly suited for personality disorders that are marked by inflexible beliefs and often linked to childhood trauma. ST emphasizes deep interaction between therapist and patient, using various exercises to explore the roots of maladaptive behaviors. To address the emotional shifts common in BPD, ST conceptualizes the patient's personality in terms of five modes: the abandoned child, angry/impulsive child, detached protector, punitive parent, and healthy adult [6,9,50]. Figure 1 illustrates the main maladaptive schemas in BPD.

Therapeutic Approach

In ST, the therapist plays the role of the “healthy parent” to nurture the patient’s inner “child” and counteract negative modes. Techniques such as limited reparenting, imagery, and cognitive exercises are used to create a supportive, parent-child dynamic, helping BPD patients navigate abandonment issues and regulate emotions. Therapy progresses through three stages: bonding and emotional regulation, mode change, and finally, autonomy development, fostering independence and self-identity [6,50].

Efficacy

Schema therapy has shown promising results in treating BPD. A randomized-controlled trial by Farrell et al. [51] found that 94% of BPD patients who received ST no longer met the diagnostic criteria after treatment compared to only 16% in the control group. Further research by Zhang et al. [52] supports ST’s effectiveness in enhancing quality of life for individuals with personality disorders.

TFP

TFP refers to a psychodynamic treatment method pioneered by Otto F. Kernberg during the 1970s and 1980s [53]. Not only does TFP focus on achieving adequate personality organization and regaining the sense of self, but it mainly focuses on the impulsive and destructive symptoms of BPD. The concept of transference is used by a therapist to closely monitoring the patient’s relationship, behavior, and attitude towards them. Transference is then analyzed in effort to make the internalized unconscious conflicts, which represent a dyad of patient-others interaction, become conscious for the patient. During this process the patient may express multiple defense mechanisms, and it is the therapist’s duty to psychoanalyze them and bring them into light, emphasizing the impact of TFP on aiming to solve the pathological causative roots of BPD, as compared to other methods of therapy such as cognitive-behavioral therapy. A transference for a BPD patient is split between positive and negative sectors. The therapist utilizes countertransference after the analysis of transference. The initial step in TFP is named “interpretation,” which basically refers to the therapist forming multiple hypothesis based on the patient’s transference and countertransference. This step is followed by transference analysis, during which the therapist should remain objective and neutral while staying outside of the patient’s activated internal conflicts. This represents a contrast to ST where the therapist deliberately inserts themselves in recalled memory exercises [50]. Finally, countertransference involves the therapist closely monitoring their own emotional responses to the patient. These reactions are used as a tool to better understand the emotional conflicts that the patient brings into the therapeutic relationship [6,54,55]. Fischer-Kern et al. [56] conducted a randomized-controlled trial of 104 BPD patients and found a significant increase in reflective function after one year of treatment with TFP.

Controversies in BPD: diagnosis, treatment, and stigmatization

The controversies surrounding BPD are multifaceted, encompassing diagnostic challenges, treatment efficacy, and the stigma associated with the disorder. Disputes often arise over the boundaries between BPD and similar psychiatric conditions, such as bipolar disorder and complex post-traumatic stress disorder (CPTSD), complicating accurate diagnosis [57,58]. Additionally, debates regarding the effectiveness of pharmacological interventions vs. psychotherapy persist, alongside concerns about the implications of labeling individuals with BPD [17,26]. Understanding these controversies is crucial for clinicians and researchers alike, as they significantly influence treatment approaches, patient experiences, and societal perceptions of BPD. This section will explore these contentious issues in greater depth.

Diagnostic challenges and early diagnosis of BPD

Ruggero et al. [57] explored the frequent misdiagnosis of BPD as bipolar disorder, showing that nearly 40% of individuals with BPD are mistakenly diagnosed with bipolar disorder at some point. This misdiagnosis often arises because both conditions share overlapping symptoms, such as mood instability, impulsivity, and intense emotional responses. However, while mood shifts in bipolar disorder typically last for days to weeks, BPD mood fluctuations are more reactive, varying within minutes or hours in response to situational triggers.

The study found that no specific symptom or criterion exclusively predicted misdiagnosis. Instead, clinicians often face diagnostic challenges due to similar emotional dysregulation and interpersonal difficulties seen in both disorders. The misdiagnosis of BPD as bipolar disorder can lead to ineffective treatment, particularly when medication prescribed for bipolar disorder fails to address the core emotional and relational symptoms associated with BPD. As a result, patients may suffer from unnecessary side effects and lack access to other therapies, such as DBT, which is tailored for BPD [57].

A study by Liu et al. [59] reached a similar conclusion that BPD shares significant symptom overlap with other psychiatric conditions, particularly mood and anxiety disorders. This overlap can lead clinicians to misattribute symptoms to more familiar or less stigmatized disorders, thus delaying an accurate diagnosis of BPD.

The study also underscores how stigma and biases within the mental health field complicate diagnosis. BPD is sometimes seen as a challenging condition to manage, which can discourage clinicians from providing this diagnosis due to fears about treatment difficulties or patient resistance. Furthermore, the symptoms of BPD, such as impulsivity, intense fear of abandonment, and turbulent relationships, can strain the clinician-patient dynamic, adding another layer of difficulty in diagnosis and treatment planning [59].

The study also highlights a gap in specialized training and diagnostic tools tailored specifically to BPD. Without adequate training and tools that clearly differentiate BPD’s unique patterns, clinicians may struggle to recognize the disorder’s distinct profile, increasing the chances of misdiagnosis [59].

Westernization of BPD criteria

The diagnostic criteria for BPD have been shaped within a predominantly Western framework, which has raised questions about their cross-cultural applicability. Western concepts of self, identity, and emotional expression, which inform these criteria, may not universally align with how personality pathology manifests across diverse cultures. Alarcón and Foulks highlighted that personality disorders cannot be fully understood without considering the cultural backdrop, as culture not only directs the expression of symptoms but also influences which behaviors are deemed pathological [60].

Cultural competency in clinical practice aims to bridge these gaps by ensuring that diagnostic frameworks are adaptable and respectful of cultural variation. This approach requires viewing culture from the individual's perspective, thereby avoiding stereotypes and overgeneralizations. For instance, Ronningstam et al. [61] emphasizes that each culture has distinct historical and social norms that influence personality functioning and emotional regulation, which are core elements of BPD. Diagnosing BPD outside of a Western context necessitates recognizing how different cultural values shape expressions of distress, especially in areas such as emotion dysregulation and interpersonal sensitivity.

Moreover, studies such as Mezzich et al. [62] have shown that culture shapes both the clinical encounter and the conceptual framework of psychiatric diagnoses. When diagnostic categories are applied across cultures without adaptation, there is a risk of cultural bias. This bias may lead to misdiagnosis or overlooking the nuanced ways in which BPD symptoms present within various cultural contexts. Cultural norms around acceptable emotional expression, relationships, and identity can influence whether a behavior is seen as indicative of personality pathology or as part of cultural norms.

Alarcón and Foulks further assert that BPD exemplifies the intersection of culture and psychopathology, where symptoms such as interpersonal instability or intense emotional responses may align with or deviate from cultural expectations, influencing both the diagnosis and experience of the disorder. In societies with communal values, for example, what might be seen as dependency or fear of abandonment in BPD could be interpreted differently due to the normative value placed on close familial and romantic bonds [62].

As globalization increases cultural diversity within societies, it is critical for clinicians to be attuned to cultural differences in symptom presentation and personality functioning. By integrating cultural awareness into diagnostic practices, mental health professionals can approach BPD not merely as a set of symptoms but as expressions shaped by a person’s cultural context. This culturally sensitive framework not only helps in more accurate diagnosis but also in creating treatment approaches that honor the patient’s cultural background, thus fostering a more humane and effective therapeutic relationship.

Etiology: the nature vs. nurture debate

The origins of BPD involve a complex interplay between genetic, biological, and environmental factors [63]. The nature vs. nurture debate explores how much BPD is influenced by hereditary traits vs. external life experiences [64]. Literature indicates that genetic predispositions can make individuals more vulnerable to BPD traits, such as emotional instability and impulsivity [64]. Meanwhile, environmental factors, especially early-life adversities, significantly shape the disorder’s development and manifestation. This interaction between genetic predisposition and environmental impact suggests that both innate and external influences contribute to BPD, challenging the notion of a singular cause [64,65].

Trauma as a Root Cause

Trauma, especially in childhood, is widely considered a foundational factor in BPD’s development. Literature consistently shows a high prevalence of adverse experiences, such as physical, emotional or sexual abuse, neglect, and inconsistent caregiving, among individuals diagnosed with BPD [66,67]. These early traumas disrupt attachment processes, emotional regulation, and self-identity formation, all of which contribute to the development of BPD’s core symptoms. While trauma is strongly linked to BPD, it is not a sole factor, as its influence is compounded by genetic and additional environmental elements, suggesting that trauma alone does not invariably lead to the disorder [68].

Is BPD a Trauma Disorder?

The substantial overlap between trauma-related symptoms and BPD features has led clinicians to potentially consider BPD a trauma-related disorder. The disorder shares notable similarities with CPTSD, including emotional dysregulation, intense relational difficulties, and fragmented identity [58,69]. The results of Lee et al. [58] categorized patients in their study population to four classes based on symptomology, where class one was named “CPTSD and BPD comorbid class,” indicating that childhood trauma associated with BPD may increase the risk for CPTSD. However, not all individuals with BPD report trauma histories, and the disorder often includes patterns of impulsivity and identity disturbance not typically associated with trauma disorders alone. This distinction suggests that while trauma can significantly influence BPD’s expression, it may not fully define its etiology, pointing instead to a multifaceted origin [69].

Pharmacological controversies and treatability

Is BPD treatable?

The perception of BPD as an untreatable condition has shifted significantly in recent years. Advances in therapeutic approaches, particularly DBT, have demonstrated that many individuals with BPD can achieve substantial symptom relief and improved functioning. DBT, developed specifically for BPD, focuses on enhancing emotional regulation and interpersonal skills. Research indicates that DBT is effective in reducing self-harm and suicidal behaviors. For instance, Linehan et al. [70] conducted a randomized-controlled trial, which found that DBT led to significant reductions in suicidal ideation compared to treatment as usual. Despite positive evidence, some clinicians remain skeptical about the long-term outcomes for individuals with BPD. They argue that while symptoms can improve, core personality traits associated with BPD may persist, complicating recovery. This skepticism can influence treatment decisions, potentially leading to underutilization of effective therapies such as DBT. Fruzzetti et al. [71] highlight the impact of family dynamics on the development and maintenance of BPD, emphasizing that maladaptive communication patterns and emotional invalidation within familial relationships can exacerbate the disorder's symptoms. They argue that these dysfunctional interactions contribute to the chronic nature of BPD, suggesting that while therapeutic interventions may alleviate certain symptoms, the core personality traits and relational difficulties associated with the disorder often endure. This perspective leads to a critical concern among clinicians: even when patients show improvement in their symptoms through evidence-based treatments, the persistence of maladaptive personality traits may complicate the recovery process. Many clinicians view BPD as inherently chronic, which can foster a belief that full recovery is unrealistic. This viewpoint is particularly prevalent among those who emphasize the role of long-standing relational patterns in shaping the symptoms of BPD. These insights underscore the importance of understanding BPD as a complex interplay of individual symptoms and family dynamics, suggesting that a comprehensive treatment approach may enhance recovery prospects for individuals navigating this challenging disorder.

Recovery vs. Remission

Recovery typically refers to a state in which an individual has achieved significant improvement in their symptoms and overall functioning, potentially to the point where the disorder no longer significantly impacts their daily life. Proponents of this perspective argue that individuals with BPD can reach a point of full recovery, characterized by stable emotional regulation, healthy interpersonal relationships, and a strong sense of self. This viewpoint is supported by evidence showing that many individuals experience substantial gains through effective therapies, particularly DBT, which promotes skills for emotional regulation and interpersonal effectiveness. Conversely, remission is often viewed as a temporary alleviation of symptoms, where individuals may experience periods of reduced symptomatology but retain the underlying personality traits and vulnerabilities associated with BPD. This perspective posits that while symptoms can improve significantly, the potential for recurrence remains, framing BPD as a chronic condition that necessitates ongoing management. Clinicians who subscribe to this view often express skepticism about the possibility of full recovery, citing the complexity of BPD and the enduring nature of certain maladaptive personality traits [71,72]. Research supports the notion that full recovery from BPD is possible for some individuals. Longitudinal studies have demonstrated that many patients experience significant improvement over time, with a substantial proportion achieving stable emotional and relational functioning [73]. These findings challenge the narrative of BPD as an unchangeable condition, suggesting that with the right interventions, individuals can achieve lasting transformation.

In conclusion, the debate between recovery and remission in BPD underscores the need for a nuanced understanding of the disorder's trajectory. While some clinicians remain cautious about the possibility of full recovery, evidence indicates that many individuals can and do achieve meaningful improvements in their lives. Fostering a recovery-oriented perspective may enhance treatment engagement and ultimately lead to better outcomes for those living with BPD.

Is Medication Effective for BPD?

The effectiveness of medication for BPD remains a topic of considerable debate. While various pharmacological agents, such as mood stabilizers, antidepressants, and atypical antipsychotics, are commonly prescribed to manage symptoms, research indicates mixed results regarding their efficacy. Lieb et al. [26] conducted a systematic review that found limited evidence supporting the use of these medications for core symptoms such as emotional dysregulation and fear of abandonment. Patients with BPD often find themselves in a state of overmedication, characterized by the concurrent prescription of multiple medications by their psychiatrists. This approach often aims to target the complex and multifaceted symptoms of the disorder. However, this raises significant concerns, including the risk of adverse effects, drug interactions, and decreased medication adherence. Individuals on multiple medications may experience heightened side effects, which can exacerbate their condition rather than improve it. Moreover, the lack of consensus on effective pharmacological treatment for BPD may contribute to this practice, indicating a need for more targeted and evidence-based approaches in prescribing [74].

## Conclusions

The evolving landscape of BPD treatment underscores the importance of integrating evidence-based therapeutic modalities while addressing the multifaceted challenges inherent to the disorder. Advances in psychodynamic treatments such as TFP and the effectiveness of DBT highlight the potential for recovery, yet the skepticism surrounding long-term outcomes remains prevalent among clinicians. The debate between recovery and remission illustrates the necessity for a comprehensive understanding of BPD, recognizing that while symptom alleviation is achievable, underlying personality traits may persist.

The diagnostic challenges posed by overlapping symptoms with other psychiatric conditions and the cultural biases in diagnostic criteria necessitate an urgent call for improved training and tools tailored specifically for BPD. Moreover, while pharmacological options are available, their effectiveness is often limited and accompanied by the risks of overmedication and inadequate symptom management. As mental health professionals navigate these complexities, a culturally sensitive, holistic approach to diagnosis and treatment will not only enhance the therapeutic relationship but also lead to better outcomes for individuals living with BPD. By fostering a recovery-oriented perspective and recognizing the significant role of family dynamics and cultural contexts, the field can move toward a more humane and effective understanding of BPD.
